# The AI-Augmented Ophthalmologist for chronic ocular diseases: a patient-centered framework for human-AI collaboration

**DOI:** 10.3389/fcell.2026.1902233

**Published:** 2026-08-18

**Authors:** Si-Rong Zhang, Shu-Yan Liu, Yu-Lin Li, Dan Ji, Guang-Yu Li

**Affiliations:** 1 Department of Ophthalmology, Second Norman Bethune Hospital of Jilin University, Changchun, China; 2 Department of Ophthalmology, Xiangya Hospital and Hunan Key Laboratory of Ophthalmology, Central South University, Changsha, China

**Keywords:** artificial intelligence, chronic ocular diseases, explainable AI, human-AI collaboration, ophthalmic workflow integration, patient-centered care

## Abstract

Artificial intelligence is increasingly positioned as a means of addressing the growing burden of ophthalmic disease, particularly in settings where specialist resources are scarce and visual impairment remains preventable. Yet much of ophthalmic AI has remained confined to algorithmic validation, with limited translation into routine care because of persistent barriers in trust, interoperability, accountability, and clinical fit. This article proposes the AI-Augmented Ophthalmologist as a conceptual and practical framework for human-AI collaboration in eye care. Rather than treating AI as a replacement for clinical expertise or as a standalone diagnostic tool, the framework defines three complementary forms of augmentation: perceptual augmentation, which extends the clinician’s capacity to interpret high-dimensional multimodal data; decisional augmentation, which supports risk prediction, treatment planning, surgical safety, and personalized management; and empathetic augmentation, which redistributes clinical capacity toward communication, shared decision-making, and patient engagement. By synthesizing evidence from clinical studies, implementation research, and emerging foundation-model applications, this review shows how AI can restructure fragmented ophthalmic workflows into a continuous, patient-centered loop spanning screening, diagnosis, treatment, and follow-up. The conceptual innovation of this framework lies in shifting ophthalmic AI from task-level automation to system-level collaboration, in which technical precision, clinical judgment, and humanistic care are integrated rather than separated. We further outline the conditions needed to move ophthalmic AI from algorithmic validation to real-world clinical integration: context-aware explainability, human-centered workflow design, adaptive regulation, continuous performance auditing, and equity-oriented deployment. The AI-Augmented Ophthalmologist offers a roadmap for moving ophthalmic AI beyond the digital showcase toward sustainable, trustworthy, and equitable clinical integration.

## 1 Introduction

Vision impairment remains a major global health challenge. At least 2.2 billion people worldwide live with near or distance vision impairment, including at least 1 billion cases that could have been prevented or remain insufficiently addressed (World Health Organization, 2019). This burden is compounded by the unequal distribution of ophthalmic expertise: ophthalmologist density has been estimated at 3.7 per million population in low-income countries, compared with 76.2 per million in high-income countries (Resnikoff et al., 2020). Chronic ocular diseases—including diabetic retinopathy, glaucoma, age-related macular degeneration, and chronic ocular surface disease—place particular pressure on this limited workforce because they require repeated assessment, treatment adjustment, and long-term follow-up.

Artificial intelligence offers a potential means of narrowing the gap between increasing clinical demand and limited specialist capacity. Ophthalmic AI can process fundus photographs, optical coherence tomography, visual fields, electronic health records, and longitudinal clinical data to support screening, diagnosis, risk prediction, treatment planning, and follow-up. Representative evidence-supported applications of AI across the chronic ocular disease care pathway are summarized in Table 1. However, high performance in controlled datasets does not necessarily translate into meaningful clinical benefit. Real-world adoption depends on external validity, workflow compatibility, professional oversight, patient acceptance, and appropriate responses to uncertain or incorrect outputs (Abràmoff et al., 2022; Tseng et al., 2021).

**TABLE 1:** Representative Evidence-Supported Applications of AI Across the Chronic Ocular Disease Care Pathway.

Representative AI application	Disease/care stage	Study design and sample	Human-AI interaction	Key clinical or patient outcome	Main limitation
AI-assisted DR grading (Xu et al., 2026)	DR; diagnosis	Paired before-after reader study; 224 images; 21 clinicians	Clinicians graded retinal images with and without AI support	CFP accuracy increased from 79.9% to 85.7% in PCPs, 81.2%–88.7% in residents, and 81.4%–88.1% in attendings	Retrospective image set; no patient-level outcomes
PathFinder OCT assistant (Fong et al., 2026)	Macular disease; diagnosis and referral	Prospective observational study; 202 eyes	Non-retina specialists used AI-assisted OCT assessment	Specificity exceeded 90%; sensitivity varied by disease and reader (0.57–0.89); median assessment time was <20 s	Single setting and device; variable sensitivity for some vision-threatening conditions
DeepDR-LLM (Li et al., 2024)	DR; diagnosis and management	Prospective implementation; unassisted PCP n = 397; PCP + AI n = 372	PCPs used integrated image- and language-based management and referral recommendations	Referable DR accuracy increased from 81.0% to 92.3%; referral adherence and self-management improved	Prospective patient implementation was single-center
OCT recurrence-prediction CADx (Jang et al., 2026)	nAMD; treatment planning	Multisession reader study; 149 eyes; 19 ophthalmologists	Specialists predicted post-treatment recurrence with staged clinical information and AI support	AI AUROC was 0.744; expert AUROCs ranged from 0.562 to 0.679; AI produced modest improvement in selected sessions	No prospective assessment of actual retreatment decisions or visual outcomes
AI-human hybrid teleophthalmology (Dow et al., 2023a)	DR; screening and referral	Prospective clinical implementation study	AI performed first-line assessment; specialists reviewed AI-positive or ungradable cases	Hybrid sensitivity 95.5%, specificity 98.2%, and gradability 95.6%	Single health system; limited long-term treatment outcomes
Real-time national AI screening (Ruamviboonsuk et al., 2022)	DR/DME; screening	Prospective multisite cohort; 7,651 patients across 9 primary-care sites	AI generated real-time referrals with retina-specialist safety over-reading	For vision-threatening DR, accuracy was 94.7%, sensitivity 91.4%, and specificity 95.4%	Performance depended on local workflow, infrastructure, and referral capacity
ACCESS autonomous AI trial (Wolf et al., 2024)	DR; screening and follow-up	Randomized controlled trial; AI n = 81; control n = 83	Autonomous point-of-care eye examination; analyzed as a distinct implementation pathway	Examination completion was 100% versus 22%; follow-through after abnormal results was 64% versus 22%	Single-center pediatric population; 6-month follow-up
E.M.M.A AI health coach (Sharma et al., 2026)	Chronic eye disease; between-visit support	Real-world pilot; 83 users; 446 chat sessions over 2 months	Patients directly used an AI health coach for information, symptom tracking, and self-management	Expert validation exceeded 95%; 88% of respondents were satisfied or very satisfied	Small uncontrolled pilot; short follow-up; 9% reported mild-to-moderate anxiety

Abbreviations: AI, artificial intelligence; CADx, computer-aided diagnosis; CFP, color fundus photography; DME, diabetic macular edema; DR, diabetic retinopathy; LLM, large language model; nAMD, neovascular age-related macular degeneration; OCT, optical coherence tomography; PCP, primary care physician.

Representative examples selected from the included evidence base. Autonomous AI is presented as a distinct implementation pathway and is not considered direct evidence of clinician-AI collaboration. Full characteristics of all included studies are provided in Supplementary Table S2.

**FIGURE 1:**
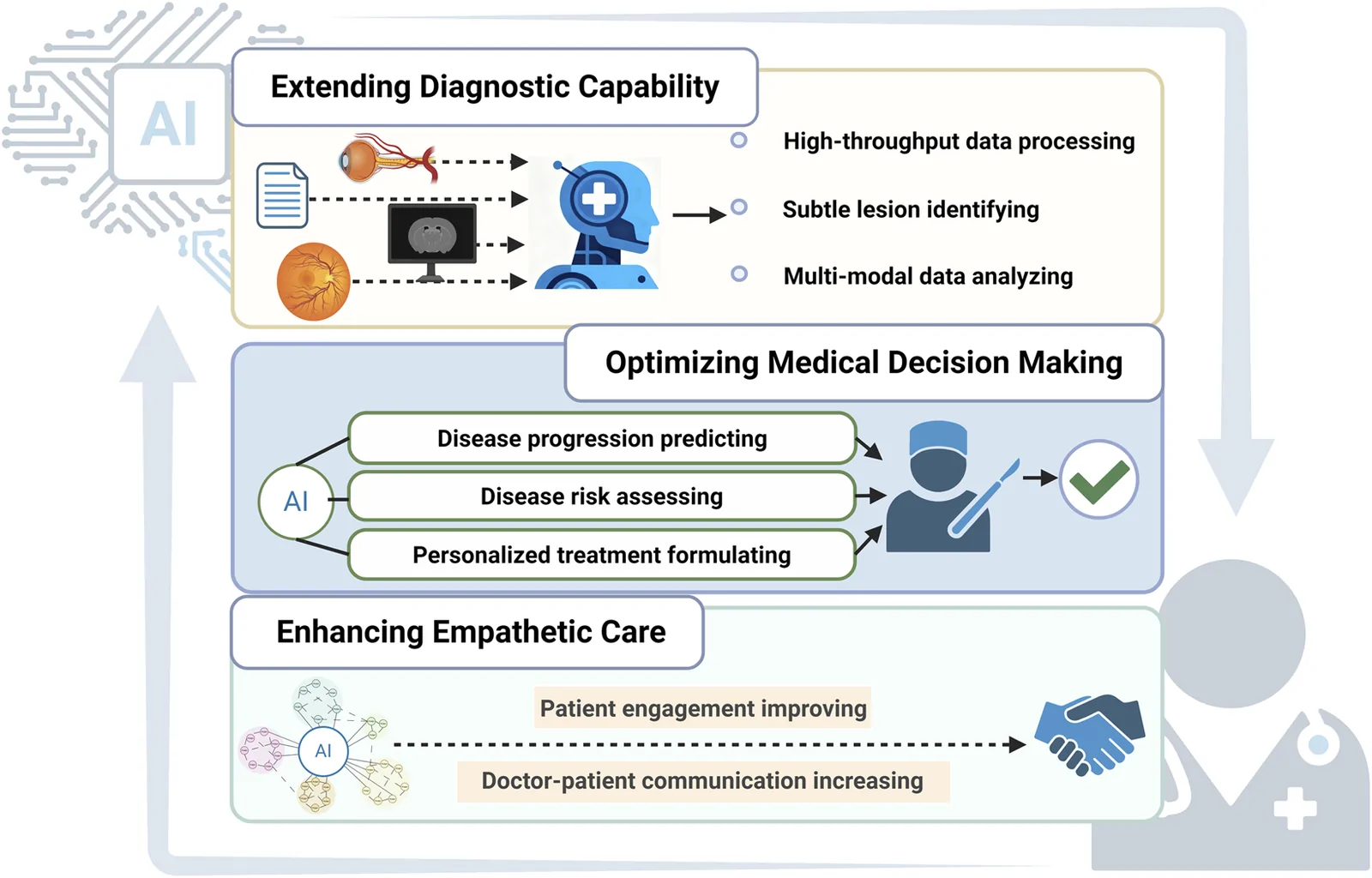
AI-Augmented Ophthalmologist. Artificial intelligence enhances the role of the ophthalmologist by augmenting their capabilities in three key areas: diagnosis, decision-making, and compassionate patient care. Created in BioRender. Zhang, S. (2026) https://BioRender.com/xcvlekn.

The key question is therefore not whether AI can replace ophthalmologists in isolated tasks, but how AI, clinicians, and patients can contribute complementary capabilities across the care pathway. As illustrated in Figure 1, AI can support high-dimensional pattern recognition and evidence synthesis, while ophthalmologists retain responsibility for contextual reasoning, ethical judgment, shared decision-making, and empathetic patient communication. Patients contribute their values, preferences, and practical circumstances. Together, these roles form the AI-Augmented Ophthalmologist, a collaborative model in which AI expands clinical capacity without displacing professional responsibility or human connection.

Against this background, we propose an evidence-informed framework comprising three related dimensions: perceptual augmentation, in which AI extends the interpretation of complex ophthalmic information; decisional augmentation, in which AI supports risk stratification, referral, treatment planning, and individualized management; and empathetic augmentation, in which reduced repetitive workload and improved information delivery may create greater capacity for communication and patient participation. Rather than representing a new technical taxonomy of AI systems, this framework provides an ophthalmologist-centered clinical perspective that integrates existing AI capabilities according to their roles in augmenting clinical expertise and supporting patient-centered care. This framework is consistent with broader calls for integrated, people-centered eye care and human-centered AI development (Chow et al., 2022; Ting et al., 2023).

This PRISMA-ScR-informed scoping review makes three contributions:

- It proposes an evidence-informed AI-Augmented Ophthalmologist framework, providing a clinical model to organize how existing AI capabilities can complement ophthalmologists’ clinical expertise and support patient-centered care.
- It maps and synthesizes current evidence on human-AI collaboration across the complete screening–diagnosis–treatment–follow-up pathway in chronic ocular diseases, while distinguishing autonomous AI from clinician-facing and human-in-the-loop systems.
- It identifies key barriers to implementation, including trust, workflow integration, accountability, lifecycle monitoring, reimbursement, and equity, and proposes a phased roadmap for safe, sustainable, and patient-centered adoption.

The AI-Augmented Ophthalmologist is therefore proposed not as a replacement for ophthalmologists, but as an organizational framework for accountable and patient-centered human-AI collaboration.

## 2 Methods

We conducted a PRISMA-ScR-informed scoping review to synthesize current evidence on human-AI collaboration in clinical ophthalmology.

### 2.1 Search strategy

A comprehensive search of three electronic databases (PubMed, Web of Science Core Collection, Scopus) was performed for literature published from 1 January 2010, to 8 July 2026. The search strategy combined three concept blocks:

1. Chronic ocular diseases;
2. Artificial intelligence technologies; and
3. Clinical implementation, human-AI interaction, or patient-centered application.

The search strategy combined three core concepts: chronic ocular diseases, artificial intelligence, and clinical or patient-centered application. Disease-related terms included diabetic retinopathy, diabetic macular edema, glaucoma, age-related macular degeneration, geographic atrophy, neovascular AMD, dry eye disease, and ocular surface disease. AI-related terms included artificial intelligence, machine learning, deep learning, large language model, ChatGPT, generative AI, foundation model, and clinical decision support. Clinical application terms included AI-assisted diagnosis, human-in-the-loop, human-AI collaboration, reader study, clinical implementation, workflow integration, risk prediction, remote monitoring, patient engagement, shared decision-making, automation bias, over-reliance, and anchoring.

Terms within each concept were combined using “OR,” and the three concept groups were combined using “AND.” The search was limited to English-language publications. Web of Science and Scopus were restricted to articles, whereas no publication-type restriction was applied in PubMed at the search stage. The complete database-specific search strategies, search dates, field restrictions, filters, and numbers of records retrieved are provided in Supplementary Table S1. All records were imported into EndNote for deduplication.

### 2.2 Study selection and eligibility criteria

Studies were included if they designed, implemented, or evaluated AI systems used in collaboration with ophthalmologists in clinical settings. We excluded purely algorithmic studies without clinical validation and studies lacking clinical implementation or evaluation. Fully autonomous AI systems were not considered evidence of direct clinician-AI collaboration; however, studies demonstrating real-world clinical implementation were retained as a separate workflow transformation pathway. Their findings were interpreted as evidence of AI-enabled care delivery rather than direct human-AI collaboration.

**FIGURE 2:**
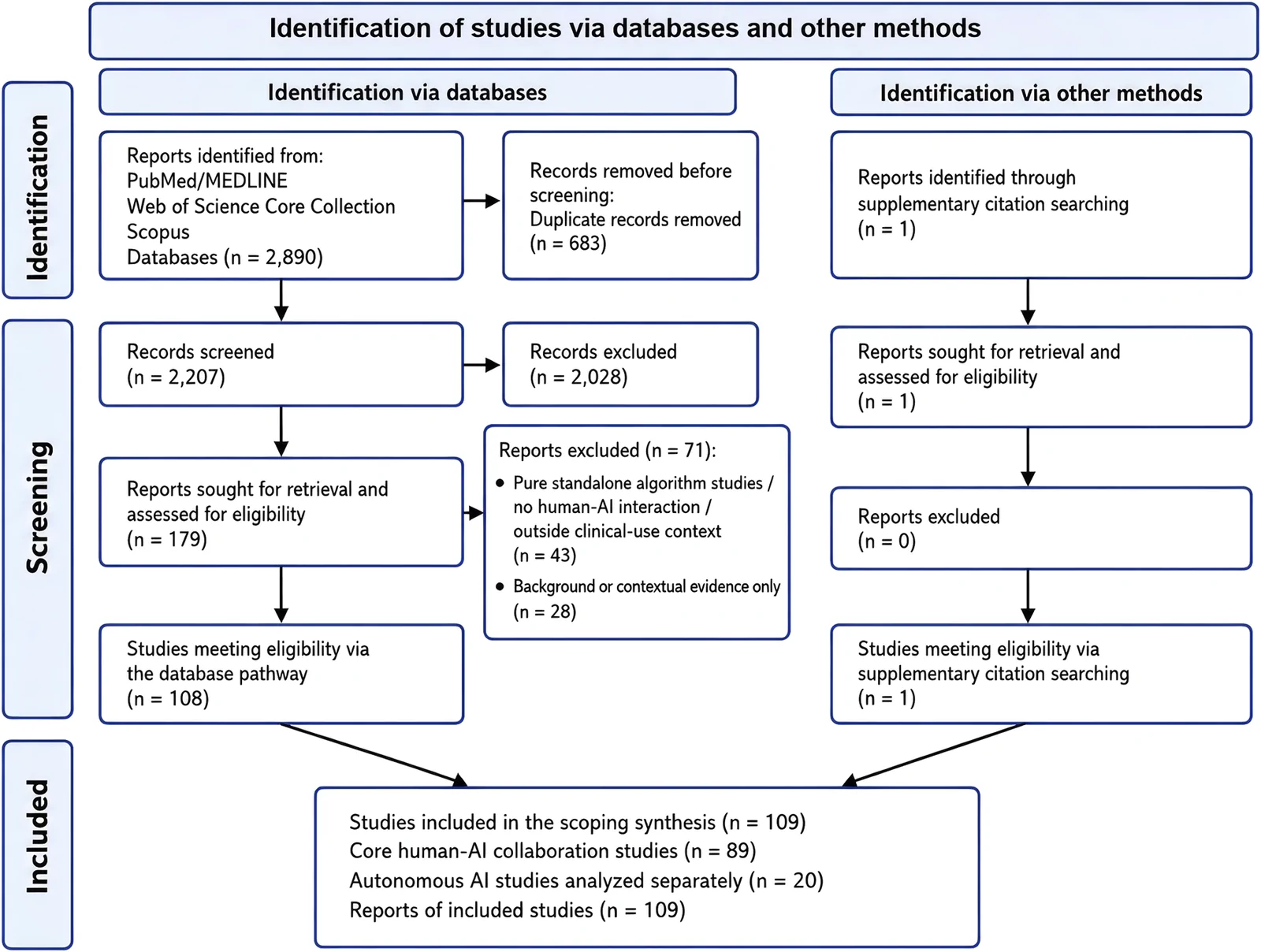
Flowchart of literature search and screening process.

The study selection process is summarized in Figure 2. A total of 2,890 records were identified, and 683 duplicate records were removed. The remaining 2,207 unique records were screened independently by two reviewers (S.R.Z. and S.Y.L.) against the eligibility criteria. The full texts of potentially relevant articles were then assessed independently by the same reviewers. Any disagreements at any stage were resolved through discussion or by consulting a third reviewer (G.Y.L.). A total of 109 reports were included in the qualitative synthesis. Among these, 89 studies evaluated clinician-facing or human-AI collaborative systems, whereas 20 studies evaluated autonomous AI workflows. Autonomous AI studies were analyzed separately as workflow transformation models rather than direct evidence of clinician-AI collaboration.

### 2.3 Data extraction and synthesis

Data were extracted using a standardized form that included study design, disease type, clinical setting, sample size, AI system, clinical task, level of autonomy, type of human-AI interaction, comparator, outcomes, main findings, and limitations. Data extraction was performed by one reviewer and verified by a second reviewer.

The included studies were grouped into clinician-facing decision support, human-in-the-loop diagnostic assistance, patient-facing AI, workflow- or system-level AI, and autonomous AI. Findings were then mapped onto the three dimensions of the AI-Augmented Ophthalmologist framework: perceptual augmentation, decisional augmentation, and empathetic or patient-centered augmentation.

Because of heterogeneity across studies, findings were synthesized narratively. Quantitative outcomes were reported as presented in the original studies and were not pooled across different clinical tasks. Greater emphasis was placed on prospective studies, reader studies, external validation studies, implementation studies, and real-world clinical deployments. Reviews and contextual publications were used only to support the Introduction and Discussion and were not included in the primary evidence synthesis. Additional contextual and technical publications were cited where relevant to support interpretation of treatment-planning applications but were not included in the formal evidence synthesis or counted in the PRISMA flow diagram.

### 2.4 Methodological quality considerations

Because the included studies covered heterogeneous designs, including reader studies, prospective implementation studies, and workflow evaluations, formal quantitative risk-of-bias pooling was not performed. Instead, methodological limitations were extracted and narratively summarized, focusing on external validation, study design, clinical setting, comparator availability, and patient-level outcomes.

## 3 The AI-Augmented Ophthalmologist: a tripartite augmentation paradigm

The core of our proposed framework is the synergistic augmentation of the ophthalmologist across three interdependent dimensions: perceptual, decisional, and empathetic. These dimensions are not intended as independent technical categories, but as clinically oriented perspectives describing different ways AI may enhance ophthalmologists’ capabilities. This framework transforms the clinician from a data processor into an enhanced decision-maker and communicator, as illustrated in Figure 1.

### 3.1 Perceptual augmentation: extending the clinician’s senses

AI can extend clinical perception by rapidly extracting disease-relevant information from fundus photographs, OCT scans, and multimodal records. The clearest evidence comes from studies in which clinicians interpreted cases with and without AI support. In a paired before-after study, AI assistance increased the diagnostic accuracy of non-ophthalmologist physicians from 82.8% to 91.1%, with particularly notable improvements in more complex diagnostic scenarios (Kim and Song, 2025). Similarly, an AI-assisted diabetic retinopathy study improved accuracy across primary care physicians, residents, and attending ophthalmologists. For color fundus photographs, accuracy increased from 79.9% to 85.7% for primary care physicians and from approximately 81%–88% for ophthalmology trainees and specialists (Xu et al., 2026).

Perceptual augmentation is most useful when it supports, rather than replaces, clinical review. In a prospective OCT referral study, non-retina specialists using the PathFinder assistant reached moderate-to-substantial diagnostic agreement with retina specialists, maintained specificity above 90%, and completed AI-assisted assessments in a median time of less than 20 s. Sensitivity nevertheless varied by disease, particularly for some vision-threatening macular conditions (Fong et al., 2026). In a reader study of an inherently interpretable model, highlighting high-evidence retinal regions improved screening speed and accuracy for difficult diabetic retinopathy decisions, while the model retained an accuracy of 0.906 and an AUC of 0.904 (Djoumessi et al., 2025). These findings indicate that AI can broaden the clinician’s effective visual field, but disease-specific failure modes and human oversight remain essential.

### 3.2 Decisional augmentation: from information to evidence-based insight

Decisional augmentation begins when AI-generated findings are translated into risk estimates, referral recommendations, or individualized management plans. An integrated image-based deep learning and language-model system increased primary care physicians’ accuracy for referable diabetic retinopathy from 81.0% without assistance to 92.3% with assistance. The same intervention was associated with better diabetes self-management and higher adherence to ophthalmic referral recommendations (Li et al., 2024). This illustrates how image interpretation and language-based guidance can be combined within a broader clinical decision pathway.

Longitudinal prediction may further shift chronic eye care from fixed schedules to risk-based follow-up. DeepDR Plus predicted time to diabetic retinopathy progression over 5 years with concordance indices of 0.754–0.846. In a simulated workflow, individualized risk assessment could extend the mean screening interval from 12.0 to 31.97 months while delaying detection of vision-threatening progression in 0.18% of participants (Dai et al., 2024). Another externally evaluated system predicted emergent referable diabetic retinopathy at 1–3 years with AUROCs of 0.91–0.93 (Nderitu et al., 2024). These models support more differentiated surveillance, but prediction accuracy should not be interpreted as proof of improved treatment outcomes. Prospective trials are still required to determine whether AI-guided decisions preserve vision, reduce unnecessary visits, and remain safe across diverse populations.

### 3.3 Empathetic augmentation: reclaiming time for humanistic care

Empathetic augmentation does not imply that AI itself provides empathy. Rather, it describes a potential redistribution of clinical work: by accelerating repetitive image interpretation, information extraction, and documentation, AI may allow ophthalmologists to devote more attention to explanation, shared decision-making, and patient concerns.

Current evidence supports time savings in several specific diagnostic tasks. In a prospective OCT referral study, non-retina specialists completed AI-assisted assessments in a median time of less than 20 s (Fong et al., 2026). An interpretable diabetic retinopathy system also improved both screening speed and accuracy for difficult cases (Djoumessi et al., 2025). More directly, a multi-institutional age-related macular degeneration (AMD) study involving 24 clinicians showed that AI assistance reduced the initial diagnostic time by 10.3 s per patient and remained 6.9–8.6 s faster in subsequent assessment rounds (Chen et al., 2025). These findings suggest that AI can reduce part of the clinician’s repetitive interpretive workload. However, the studies did not directly evaluate whether the saved time was subsequently used for longer consultations or more empathetic communication.

Time efficiency alone is therefore insufficient; the released clinical capacity must be deliberately redirected toward patient-centered interaction. Current communication-oriented studies mainly examine whether large language models can improve consultation quality, patient education, and access to understandable clinical information. ChatGPT-4o assistance improved the coherence, comprehensiveness, and safety of ophthalmologists’ responses to complex clinical cases; however, approximately 44% of the additional citations generated with assistance were hallucinated, nonexistent, or incorrect (Inooka et al., 2026). This dual effect illustrates why generative AI may support communication but cannot replace professional verification.

Patient-facing studies show similar opportunities and limitations. In dry eye education, Gemini achieved a mean understandability score of 73.4 compared with 52.5 for conventional American Academy of Ophthalmology materials, while ChatGPT achieved the highest mean content-accuracy score among the tested chatbots (Soysal et al., 2026). Other analyses found substantial differences in cultural sensitivity, response depth, and delivery time across language models (Wu et al., 2025). In glaucoma, AI-generated summaries were fully correct in 66%–70% of clinic notes, although targeted extraction of diagnosis, progression, and treatment information reached 80%–91% accuracy (Zhang et al., 2026).

Together, these findings support a two-step model of empathetic augmentation: AI may first reduce repetitive cognitive and documentation burdens, and may then support clearer explanations and continuity of care. The ophthalmologist remains responsible for converting this additional capacity into attention to patient values, concerns, and preferences.

## 4 Current state of AI in clinical ophthalmic practice

**FIGURE 3:**
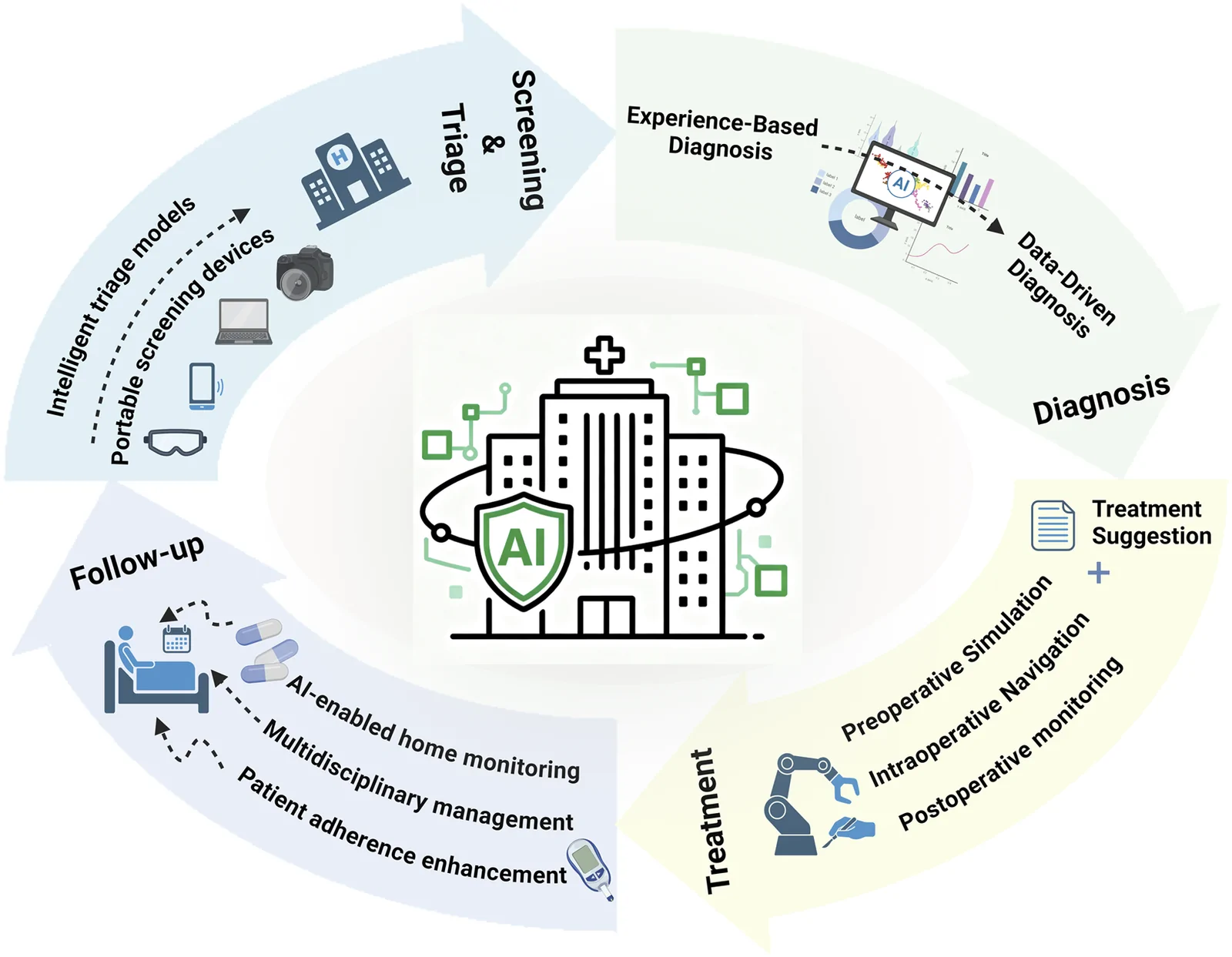
AI-established intelligent closed-loop system for clinical workflows. AI supports four connected stages of care: screening and triage, multimodal diagnosis, treatment planning and intervention, and longitudinal management and follow-up. This transforms traditionally fragmented and isolated components into an intelligent, patient-centric closed-loop system encompassing prevention, diagnosis, treatment, and follow-up. Created in BioRender. Zhang, S. (2026) https://BioRender.com/6vorsma.

Traditional ophthalmic workflows have long been hampered by a “fragmented silo” effect—where screening, diagnosis, treatment, and follow-up stages lack effective integration. This leads to a significant portion of high-risk patients (e.g., those with progressive glaucoma) missing critical intervention opportunities due to loss to follow-up. The integration of artificial intelligence aims not merely to enhance the efficiency of individual steps but to enable a holistic redesign of the entire “screening-diagnosis-treatment-follow-up” pathway. This constructs a closed-loop system characterized by “precise reach, intelligent decision-making, real-time intervention, and dynamic management” (Figure 3), which may contribute to redefining efficiency and the boundaries of precision in ophthalmic care. Unless otherwise specified, evidence discussed below refers primarily to clinician-facing or human-in-the-loop AI systems. Autonomous AI applications are interpreted separately as workflow transformation models rather than direct human-AI collaboration.

### 4.1 AI-enhanced screening and triage

Screening is the most mature clinical application of ophthalmic AI. In a prospective national programme in Thailand, a real-time deep learning system evaluated 7,651 eligible patients across nine primary care sites. For vision-threatening diabetic retinopathy, it achieved an accuracy of 94.7%, sensitivity of 91.4%, and specificity of 95.4%, with performance comparable to retina-specialist over-reading (Ruamviboonsuk et al., 2022). Similar real-world studies have shown that portable fundus cameras and offline AI can extend screening to community, rural, endocrinology, and primary care settings, although image quality and downstream referral capacity remain important constraints.

The main value of point-of-care AI may lie in converting screening results into timely action. In the ACCESS randomized trial, diabetic eye examination completion among youth was 100% with autonomous AI at the diabetes visit compared with 22% after conventional referral and education. Among participants with abnormal results, 64% completed follow-through with an eye-care provider, compared with 22% in the control group (Wolf et al., 2024). In Rwanda, immediate communication of AI-supported referral results increased referral attendance from 39.6% to 51.5% compared with delayed human grading (Mathenge et al., 2022). These trials show that rapid feedback and integration into an existing visit can improve completion of the care pathway, not merely diagnostic accuracy.

Autonomous AI should nevertheless be distinguished from human-AI collaboration. In teleophthalmology, standalone AI achieved high sensitivity for more-than-mild diabetic retinopathy but lower specificity and gradability than retina specialists. A two-step workflow in which specialists reviewed AI-positive or ungradable cases preserved sensitivity at 95.5%, increased specificity to 98.2%, and improved gradability from 63.5% to 95.6% (Dow et al., 2023a). This hybrid model illustrates a practical division of labor: AI performs rapid first-line assessment, while clinicians resolve uncertainty, confirm referrals, and manage complex cases.

This model has the potential to transform how ophthalmic care is delivered, promoting the reallocation of high-quality resources to the community and effecting a fundamental shift from “patients traveling to the hospital” to “services proactively reaching patients.” It effectively extends the “last mile” of ophthalmic service, offering a scalable solution for medically underserved areas. However, most successful cases remain confined to tightly controlled research environments or demonstration projects led by elite teams. Model performance is highly dependent on training data quality, and integration into primary care workflows requires reshaping existing clinical processes and staffing configurations.

### 4.2 Multimodal data fusion-assisted ocular disease diagnosis

The evolution of the diagnostic paradigm is a core component of workflow restructuring. Multimodal AI seeks to reproduce the integrative reasoning used by ophthalmologists by combining complementary imaging findings, clinical risk factors, and contextual information. Its principal value is not simply to improve a single classification score, but to provide a broader representation of disease severity, associated complications, and referral need.

In a community-hospital study of 600 patients with diabetes, fundus-based AI detected referable diabetic retinopathy with a sensitivity of 97.8%, specificity of 98.4%, and AUC of 0.981, while the OCT component detected macular edema with a sensitivity of 91.3%, specificity of 97.5%, and AUC of 0.944. Adding OCT increased the number of patients identified for referral from 45 to 75 by ophthalmologists and from 53 to 85 by the AI system, demonstrating the value of complementary imaging for more complete referral decisions (Liu et al., 2022).

DeepDR-LLM further combined retinal image analysis with medical history, physical examination, and laboratory data. With AI assistance, primary care physicians’ accuracy for referable diabetic retinopathy increased from 81.0% to 92.3%, while the median assessment time decreased from 14.66 to 11.31 s per eye. In prospective implementation, AI-supported care was also associated with improved self-management and greater adherence to ophthalmic referral recommendations (Li et al., 2024).

Multimodal models have also shown value in longitudinal risk prediction. In an external validation study, combined retinal images and clinical variables achieved AUROCs of 0.93, 0.93, and 0.91 for predicting emergent referable diabetic retinopathy at one, two, and 3 years, respectively (Nderitu et al., 2024).

Together, these findings suggest that multimodal AI can enhance ophthalmologists’ diagnostic capacity by identifying complementary abnormalities, integrating ocular and systemic information, and supporting referral or follow-up decisions. This shift from an experience-only reasoning toward data-informed clinical reasoning, not only enhances diagnostic precision but also enables early risk warning for disease progression. However, its clinical value depends on whether the added modality provides meaningful information beyond that available from a single data source.

### 4.3 AI-assisted treatment planning and intervention

The next stage of ophthalmic AI is continuous, risk-based management rather than one-time detection. In chronic ocular disease management, AI-assisted treatment planning focuses primarily on timing, personalization, and monitoring of interventions rather than autonomous treatment delivery.

At the treatment stage, AI may assist ophthalmologists in determining retreatment timing, selecting among therapeutic options, and identifying patients who may require escalation of intervention. In neovascular age-related macular degeneration, an AI recurrence model evaluated in 149 eyes with 19 ophthalmologists achieved an AUROC of 0.744, compared with mean expert AUROCs ranging from 0.562 to 0.679 across different information conditions. AI-supported decisions showed a modest improvement in predicting recurrence within 3 months, indicating potential value in planning anti-VEGF retreatment (Jang et al., 2026).

AI may also support individualized treatment intervals and agent selection. In post-hoc analyses of 205 ARIES and 112 ALTAIR participants, models using early OCT data predicted whether the next aflibercept interval should be shorter or longer than 12 weeks, with AUCs of 0.87 and 0.78, respectively; prediction of individual injection frequency achieved AUCs of 0.78–0.84 (Gutfleisch et al., 2025). Another model generated agent-specific post-treatment OCT predictions for ranibizumab and aflibercept, with different predicted fluid outcomes in approximately 18.5% of cases, suggesting a possible role in individualized drug selection (Moon et al., 2023).

For glaucoma, a multimodal model incorporating clinical history, medications, OCT, and visual-field measurements predicted the need for surgical intervention at one, two, and 3 years with AUCs of 0.93, 0.92, and 0.93, respectively (Christopher et al., 2024). Such predictions may support earlier recognition of inadequate disease control and more timely escalation from medical or laser treatment to surgery. Overall, current treatment-stage AI should be regarded as decision support for treatment timing, intensity, and modality rather than as an autonomous prescribing system.

### 4.4 AI-assisted chronic disease management

The next stage of ophthalmic AI is continuous, risk-based management rather than one-time detection.

DeepDR Plus predicted diabetic retinopathy progression over 5 years with concordance indices of 0.754–0.846. In a simulated workflow, individualized risk assessment increased the mean screening interval from 12.0 to 31.97 months, while delayed detection of vision-threatening progression occurred in 0.18% of participants (Dai et al., 2024). Another externally validated model achieved AUROCs of 0.93, 0.93, and 0.91 for predicting emergent referable diabetic retinopathy at one, two, and 3 years, respectively (Nderitu et al., 2024). DeepDR Plus was developed and validated using large, multiethnic longitudinal datasets, although its proposed extension of screening intervals was based on workflow simulation rather than a prospective management trial.

In glaucoma, GenVF generated explainable forecasts of future visual-field appearance for up to 10 years. In a reader study involving eight ophthalmology residents and three faculty graders, AI forecasts increased residents’ confidence and produced more conservative progression assessments, although overall diagnostic error changed only modestly and reaction times were comparable with and without AI (Tian et al., 2026). These findings suggest that longitudinal AI may support progression review and clinical education, but its effect varies according to clinician experience and individual patient characteristics.

AI-enabled workflows may also improve continuity after screening. In a programme involving 2,243 adults with diabetes, 69.2% of patients with AI-detected more-than-mild diabetic retinopathy attended ophthalmic follow-up within 90 days. Among patients attending the university eye institute, follow-up was 35.5% under the AI workflow compared with approximately 12% under human or hybrid store-and-forward workflows, potentially reflecting the faster availability of AI results within 48 h (Dow et al., 2023b).

Patient-facing AI may further extend support between visits. In a pilot study of the E.M.M.A health coach, 83 patients generated 446 chat sessions over 2 months; 88% of survey respondents were satisfied or very satisfied, and expert validation exceeded 95%, although 9% reported mild-to-moderate anxiety (Sharma et al., 2026). GlaucomaGuide achieved a mean evaluation score of 2.89, compared with 2.53 for ChatGPT-4 and 2.70 for Gemini, and successfully resolved 75% of complex patient scenarios requiring multi-turn contextual dialogue (Raipal and Penkova, 2026). Patient-facing AI applications remain exploratory. For example, independent evaluations of glaucoma-oriented conversational systems have demonstrated potential for contextual question answering; however, these studies primarily assess information delivery rather than clinical outcomes.

Together, these studies suggest that AI may support chronic ocular disease care through individualized surveillance, longitudinal progression assessment, faster referral feedback, and between-visit patient support. However, evidence for sustained adherence, visual outcomes, treatment modification, and long-term safety remains preliminary.

## 5 The significance of AI-assisted diagnosis and treatment: patient-centered care

**FIGURE 4:**
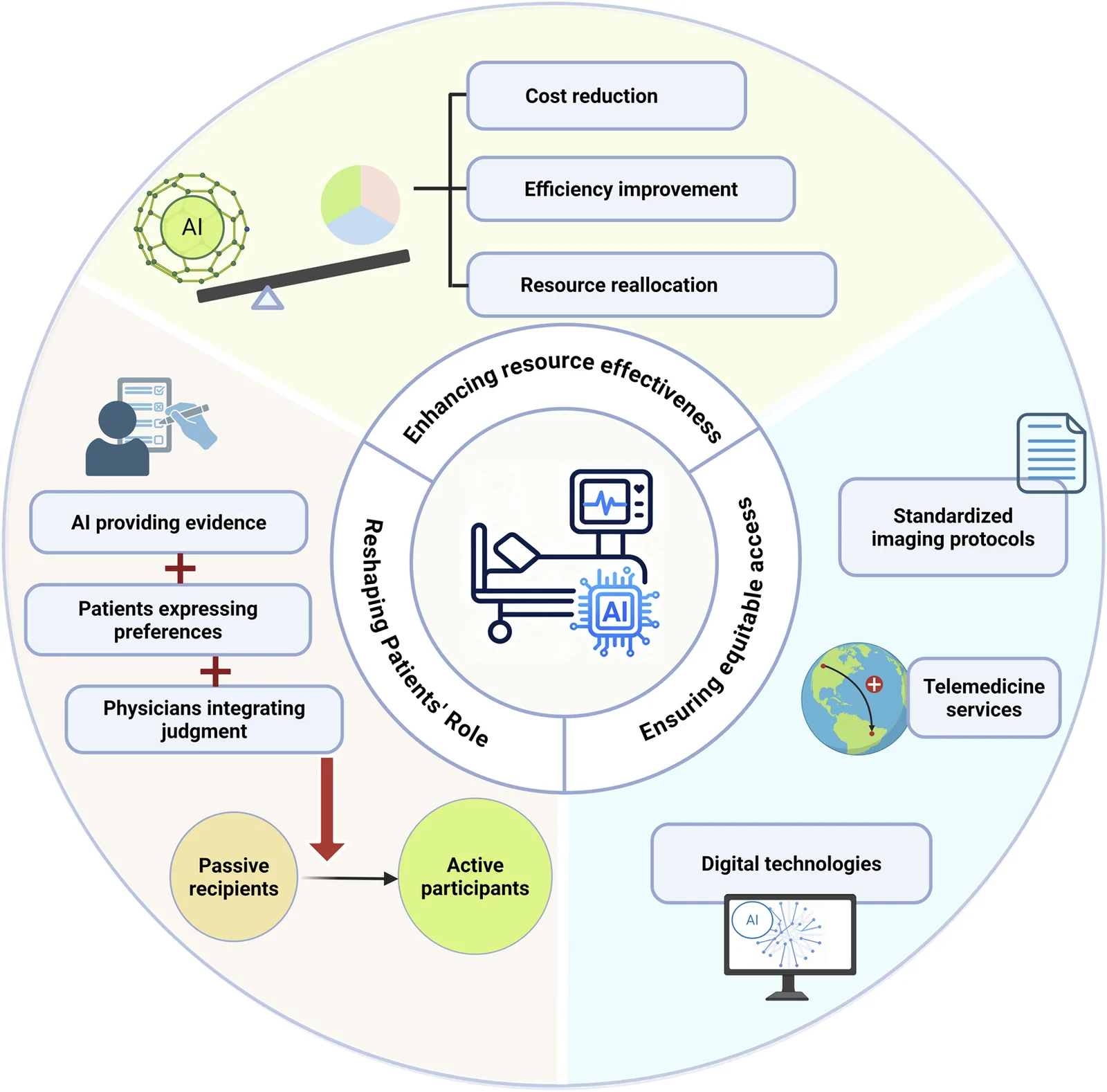
A Tripartite Pathway to Patient-Centered Intelligent ophthalmic care. Artificial intelligence establishes a new paradigm for patient-centered eye health services through a tripartite pathway: enhancing resource effectiveness, redefining patients’ roles in the health management, and ensuring equitable access through fairness-by-design technology. Created in BioRender. Zhang, S. (2026) https://BioRender.com/pr96ic9.

The ultimate value of AI in ophthalmology extends beyond improving diagnostic and therapeutic efficiency. More profoundly, it catalyzes a shift from a disease-centered to a patient-centered model of care. This shift is crucial for bridging global equity gaps in ophthalmic services. The potential of AI lies not in merely optimizing existing workflows but in achieving genuine health equity through patient empowerment and systemic transformation. This system-level change can be realized via a tripartite pathway: resource optimization, patient empowerment, and fairness-by-design in technology (Figure 4).

### 5.1 AI-optimized healthcare resource allocation

The ‘amplifying effect’ on healthcare resources is particularly pronounced within tiered diagnosis and treatment systems. Its core principle leverages technology to extend high-quality resources to the primary care level. AI may reshape ophthalmic resource allocation by redistributing specialist-level capabilities across different levels of the healthcare system. Rather than requiring every patient to attend a tertiary eye center for initial assessment, AI-enabled imaging and decision-support tools can embed standardized screening, preliminary diagnosis, and referral guidance within primary care, community clinics, endocrinology services, and remote settings. This allows ophthalmologists to concentrate on patients with uncertain findings, vision-threatening disease, or complex treatment needs.

Real-world studies demonstrate this decentralization of care. In a prospective national programme in Thailand, a real-time deep learning system was deployed across nine primary care sites and screened 7,651 eligible patients, achieving performance comparable to retina-specialist over-reading for vision-threatening diabetic retinopathy (Ruamviboonsuk et al., 2022). In Northern Ontario, autonomous AI screening was successfully completed in 93.6% of participants within primary care, and 76.0% of patients with referable disease subsequently attended ophthalmic assessment (Bhambhwani et al., 2025). These models illustrate how diagnostic capacity can be transferred closer to patients while preserving specialist referral for those most likely to benefit.

AI may also support redistribution of clinical decision-making, not merely image grading. Integrated image and language models have improved non-specialist physicians’ recognition of referable diabetic retinopathy and provided management and referral recommendations based on retinal findings and systemic clinical information (Li et al., 2024). Hybrid workflows further allow AI to perform first-line assessment while ophthalmologists review AI-positive, ungradable, or clinically discordant cases. In teleophthalmology, specialist review of selected AI outputs increased specificity to 98.2% and gradability to 95.6%, demonstrating how human and technological resources can be assigned according to case complexity (Dow et al., 2023a).

Timely AI-generated results can additionally improve the allocation of referral capacity. Immediate feedback increased referral attendance in Rwanda from 39.6% to 51.5%, suggesting that the value of decentralized screening depends on whether patients are effectively connected to specialist care (Mathenge et al., 2022). Thus, AI-enabled resource reallocation should be understood as a coordinated pathway: routine screening and preliminary assessment are shifted toward community settings, non-specialists receive structured decision support, and ophthalmologists focus their time on confirmation, treatment planning, and complex disease management. This resource allocation logic—“enabling experts to focus on what experts do best”—is redefining the cost-effectiveness ratio of ophthalmic services.

### 5.2 AI-reshaped patient role

The transformation of the patient’s role is central to patient-centered care. AI can shift patients from passive recipients of screening results toward active participants in follow-up, self-management, and clinical decision-making. In a primary-care pilot in Northern Ontario, autonomous AI screening was successfully completed in 93.6% of 202 participants. Among patients with referable disease, 76.0% attended an ophthalmology appointment, and satisfaction with integrating the eye examination into the primary-care visit averaged 4.8 out of 5 (Bhambhwani et al., 2025). The ACCESS and RAIDERS trials similarly showed that immediate AI-generated results can improve examination completion and referral uptake (Wolf et al., 2024; Mathenge et al., 2022).

Patient education and communication tools may further support participation by translating clinical information into accessible language. Chatbots, AI-generated visit summaries, and health coaches can provide repeated explanations outside the consultation, clarify treatment options, and help patients formulate questions. Together, these applications support a three-way model of shared decision-making in which AI provides structured evidence, patients articulate their preferences and practical constraints, and physicians integrate these inputs with clinical judgment. This shifts the patient from a passive recipient toward a co-creator of care, while preserving the physician’s responsibility for final clinical decisions.

However, access to information does not automatically result in meaningful participation. Current systems may omit clinical context, generate incorrect references, or provide only partially accurate summaries. Acceptance studies also indicate that perceived usefulness and ease of use influence willingness to adopt AI, whereas trust alone does not guarantee engagement (Jin et al., 2025). Patient-centered implementation therefore requires transparent explanations, informed consent, accessible non-AI alternatives, and straightforward pathways to human review.

### 5.3 AI-driven technological equity and accessibility

Equity in AI-enabled ophthalmic care extends beyond the geographic availability of a screening device. It concerns whether underserved populations can access the complete care pathway, whether AI systems perform reliably across different populations and clinical environments, and whether patients retain meaningful agency in decisions arising from AI-generated information.

At the level of access, portable imaging, teleophthalmology, and locally deployable AI can extend screening to primary-care and resource-limited settings. In Rwanda, AI-based diabetic retinopathy screening among 827 participants achieved a sensitivity of 92% and specificity of 85%, with 99.5% reporting satisfaction. However, referral adherence among those referred for diabetic retinopathy was only 53.4%, showing that access to screening does not necessarily ensure access to specialist assessment and treatment (Whitestone et al., 2024). Immediate communication of AI-generated results increased referral attendance from 39.6% to 51.5% compared with delayed human grading, further indicating that equitable access depends on how effectively screening is connected to downstream care (Mathenge et al., 2022).

Equity must also be addressed at the level of technical representation and local applicability. In Australian endocrinology and Aboriginal Medical Services clinics, AI-assisted screening achieved an AUC of 0.92, sensitivity of 96.9%, and specificity of 87.7%, and more than 93% of participants reported satisfaction and willingness to use the service again. Nevertheless, 14% of consenting participants were excluded from the primary analysis because of ungradable or missing images, commonly related to small pupils and cataract (Scheetz et al., 2021). These findings illustrate why representative datasets, affordable and adaptable devices, standardized image acquisition, local validation, and transparent reporting of ungradable cases are necessary to prevent AI from reproducing existing disparities.

Moreover, technological availability is insufficient when patients cannot complete the subsequent care pathway. In a pragmatic Indian primary-care study involving 600 participants, referral adherence remained only 13%–17% across facility-based screening, community AI-assisted screening, and standard care. Financial limitations, transportation difficulties, limited awareness, and perceived good eye health were major barriers (Chauhan et al., 2025). Equity therefore requires not only deployment of AI in underserved locations, but also accessible counseling, referral navigation, affordable confirmatory care, and sufficient specialist capacity.

A further dimension is relational equity—the distribution of information and participatory power within clinical decision-making. In an equitable AI-supported pathway, AI provides structured evidence, patients articulate their preferences, values, and practical constraints, and physicians integrate these inputs with clinical judgment. Patient participation, however, cannot be assumed simply because AI-generated information is available. Acceptance research suggests that perceived usefulness and ease of use influence patients’ willingness to adopt AI-supported diabetic retinopathy screening, whereas trust alone may not ensure engagement (Jin et al., 2025). AI should therefore strengthen informed participation rather than introduce a new form of technological authority, while physicians retain responsibility for interpretation, safety, and final clinical decisions.

Together, these dimensions suggest that the equity of ophthalmic AI should be evaluated through three questions: who can access the complete AI-enabled care pathway, for whom the technology performs reliably, and who is meaningfully empowered to participate in the resulting decisions.

## 6 Real-world challenges for AI in ophthalmic care

**FIGURE 5:**
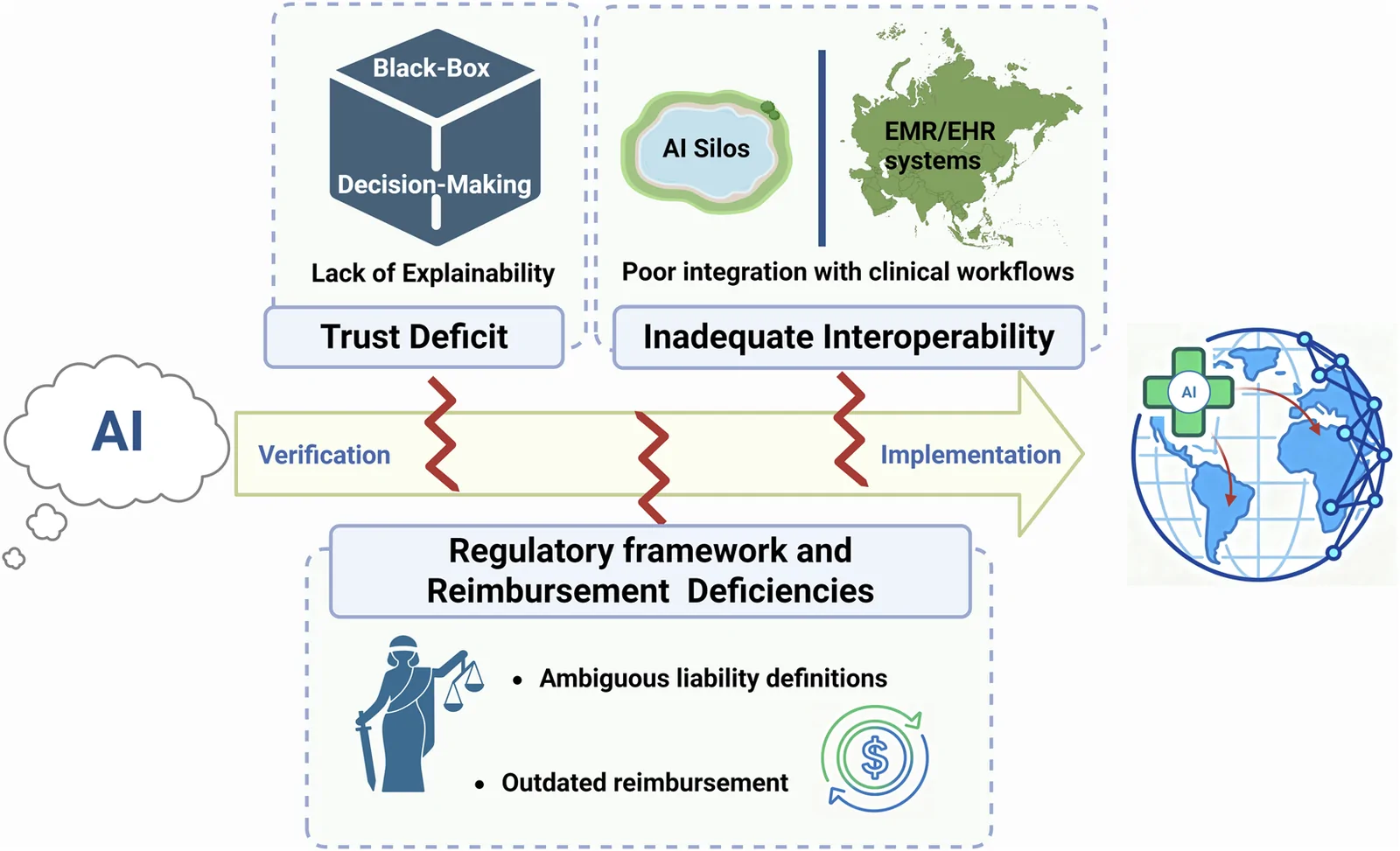
Real-world challenges in ophthalmic AI. Trust deficits, inadequate interoperability, and ambiguous accountability and reimbursement models as key barriers to widespread adoption. Created in BioRender. Zhang, S. (2026) https://BioRender.com/h1h8dcm.

Despite demonstrating significant potential, the real-world clinical application rate of artificial intelligence in ophthalmology remains suboptimal. This chasm between technical promise and clinical deployment stems from four systemic barriers: technical trust, workflow integration, dynamic regulation, and reimbursement mechanisms (Figure 5). Bridging this gap requires integrated, multi-dimensional solutions and a structured, phased implementation roadmap.

### 6.1 The trust deficit

Trust in ophthalmic AI depends not only on model performance, but also on the quality of the evidence used to develop, validate, and explain its outputs. Interpretable diabetic retinopathy models can highlight clinically relevant retinal features and improve clinicians’ speed and accuracy in difficult screening cases, suggesting that appropriately designed explanations may support clinical interpretation (Djoumessi et al., 2025).

Interpretability, however, does not guarantee correctness. In a nationwide analysis of 736,083 retinal images, adjudication of disagreements revealed an estimated reference-label error rate of only 1.2%. Nevertheless, correcting these labels increased the estimated sensitivity of the deep learning system from 79.6% to 92.5% (Wang et al., 2024). This finding shows that apparent model errors may sometimes reflect limitations in the reference standard rather than the algorithm alone. Reliable evaluation therefore requires transparent adjudication procedures and careful interpretation of disagreement between AI and clinicians.

Generative AI introduces a different set of trust-related concerns. Large language model assistance improved the organization, completeness, and perceived safety of ophthalmologists’ responses to complex cases, but also generated inaccurate or nonexistent citations (Inooka et al., 2026). Similarly, patients and practitioners involved in the English NHS diabetic eye screening programme generally anticipated improvements in efficiency, while expressing concerns about accuracy, accountability, and the need for appropriate education (Wahlich et al., 2025).

Trust should therefore be calibrated rather than assumed or maximized. AI systems should communicate uncertainty clearly, provide access to the clinical evidence underlying their outputs, and allow clinicians to accept, question, or override recommendations. When an AI output is uncertain, clinically implausible, or inconsistent with other findings, the system should facilitate specialist review rather than encourage automatic acceptance. This is particularly important because plausible explanations and confident language may promote anchoring, automation bias, and over-reliance even when the underlying recommendation is incorrect.

### 6.2 Insufficient interoperability

Clinical integration requires more than placing an AI algorithm alongside an imaging device. AI outputs must be incorporated into a coordinated pathway that connects patient identification, image acquisition, result review, referral, specialist confirmation, treatment, and longitudinal follow-up. The key implementation challenge is therefore not simply whether the model produces an accurate result, but whether that result reaches the appropriate clinician or patient and leads to a clearly assigned clinical action.

This distinction is illustrated by an implementation study that transformed a non-digital screening service into a cloud-based pathway integrating AI grading, remote specialist over-reading, and referral tracking. During AI-assisted screening periods, 645 patients were evaluated, and 89.1% of those with confirmed referable disease completed referral. Specialist review also detected a 1.1% false-negative rate among cases not referred by the AI system (Chotcomwongse et al., 2025). The clinical value of the programme therefore arose from the integration of AI with human review and referral management, rather than from automated grading alone.

Hybrid workflows further demonstrate the importance of explicit exception handling. In teleophthalmology, directing AI-positive and ungradable cases to specialist review improved the reliability of the overall pathway (Dow et al., 2023a). This model assigns routine image assessment to AI while reserving ambiguous, poor-quality, or clinically discordant cases for ophthalmologists, thereby defining how uncertainty should move through the system.

Conversely, real-world autonomous screening in an endocrinology clinic generated definitive results for 95.7% of participants, yet only 44.7% of patients with positive findings completed confirmatory examination within the same institution (Gil et al., 2026). This gap highlights the difference between completing an AI test and completing a clinical care pathway.

Effective integration therefore requires interoperable data transfer, clear assignment of referral ownership, mechanisms for reviewing ungradable or discordant results, timely communication with patients, and documentation of subsequent clinical actions. Without these components, AI may produce technically valid outputs that remain disconnected from diagnosis, treatment, and follow-up.

### 6.3 Ambiguous accountability and reimbursement mechanisms

Accountability must be defined across the entire AI-enabled care pathway rather than assigned only at the point of algorithmic output. For autonomous systems, healthcare organizations should specify who is responsible for image acquisition, communication of results, management of ungradable cases, referral initiation, and follow-up of patients with positive findings. For clinician-facing systems, the AI output, the clinician’s final decision, and any clinically important disagreement should be documented. Responsibility should therefore be distributed explicitly among developers, healthcare institutions, and clinicians, while final patient-care decisions remain under professional and organizational oversight. Broader governance guidance similarly emphasizes human autonomy, transparency, accountability, and responsiveness throughout the use of AI in healthcare (World Health Organization, 2021).

Predeployment validation alone is insufficient because model performance may change after implementation. Differences in cameras, image quality, disease prevalence, patient demographics, clinical workflows, and software updates may alter performance over time. Post-deployment monitoring should therefore assess gradability, false-negative and false-positive rates, subgroup performance, clinician overrides, referral completion, and patient outcomes at each implementation site. Changes to the model or its intended use should also be documented and re-evaluated. Current regulatory guidance increasingly adopts this total-product-lifecycle approach and recommends planned procedures for monitoring deployed models and managing subsequent modifications (U.S. Food and Drug Administration, 2021; U.S. Food and Drug Administration, 2024).

Reimbursement represents a related implementation challenge because the value of AI is generated by the complete care pathway rather than by the automated test alone. In Australia, population modelling suggested that broad implementation of AI-based diabetic retinopathy screening could increase detection, prevent blindness, gain quality-adjusted life-years, and generate long-term health-system savings (Hu et al., 2024). In contrast, an analysis based on real-world data from urban China found that AI-based community screening was not cost-effective under baseline assumptions and became favorable only when referral adherence increased or screening costs decreased (Lin et al., 2023).

These findings suggest that reimbursement and procurement models should consider not only image analysis, but also image acquisition, patient communication, specialist review, confirmatory examination, referral navigation, treatment access, system maintenance, and ongoing safety monitoring. Without clearly assigned responsibility, lifecycle oversight, and support for downstream care, AI risks becoming a technically accurate but clinically disconnected service.

## 7 Future directions for AI in clinical ophthalmology

**FIGURE 6:**
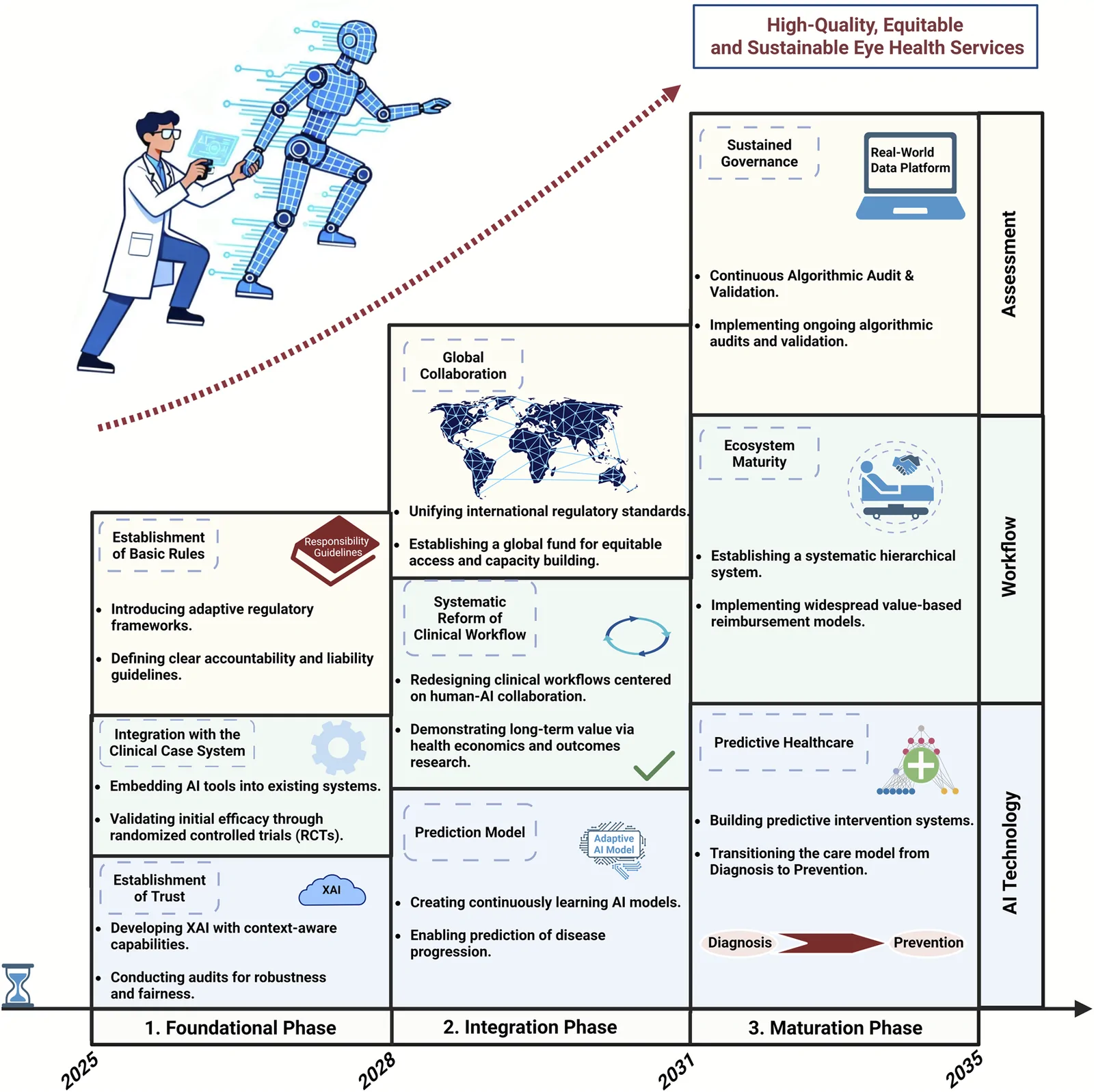
Ophthalmic AI integration roadmap (2025–2035). This roadmap outlines the concurrent advancement and spiral development across three critical dimensions: technological, workflow, and regulatory. The technology pathway progresses from developing trustworthy explainable AI (XAI) and conducting robustness and fairness audits, to creating continuously learning models and enabling prediction of disease progression, culminating in building predictive intervention systems and transitioning the care model from diagnosis to prevention. The workflow pathway advances from embedding AI tools into existing systems and validating initial efficacy through randomized controlled trials (RCTs), to redesigning clinical workflows centered on human-AI collaboration and demonstrating long-term value via health economics and outcomes research, finally establishing a systematic hierarchical system and implementing widespread value-based reimbursement models. The regulatory pathway moves from introducing adaptive regulatory frameworks and defining clear accountability guidelines, to unifying international regulatory standards and establishing a global fund for equitable access and capacity building, ultimately ensuring continuous monitoring of AI performance and implementing ongoing algorithmic audits and validation. Through continued collaboration and iterative progress, we aim to establish a comprehensive, patient-centered paradigm of human-AI collaboration in ophthalmic care by 2035. The timeline represents a proposed conceptual roadmap rather than a predictive forecast. Created in BioRender. Zhang, S. (2026) https://BioRender.com/psejc88.

The challenges identified in Section 6—including insufficiently calibrated trust, fragmented workflows, unclear accountability, and limited evidence of long-term clinical benefit—define the priorities for the next stage of ophthalmic AI. Future development should therefore move beyond isolated improvements in algorithmic performance and focus on the translation of AI into safe, patient-centered, and sustainable clinical care. As illustrated in Figure 6, we propose a three-phase roadmap for the development of AI-augmented ophthalmology from 2025 to 2035.

*From Algorithmic Accuracy to Trustworthy Human-AI Teams: Strengthening Clinical Evidence:* The first priority is to establish reliable evidence for clinicians and AI working together. Future research should progress from retrospective accuracy studies toward prospective, multisite evaluations conducted under realistic clinical conditions. These studies should account for time pressure, variable image quality, incomplete clinical information, and situations in which AI recommendations are uncertain or incorrect.

Evaluation should extend beyond sensitivity, specificity, and area under the curve to include diagnostic calibration, clinician workload, referral completion, treatment modification, visual outcomes, adherence, quality of life, and unintended harms. Explainable AI should similarly move beyond generic heatmaps by providing clinically meaningful evidence, communicating uncertainty, and adapting explanations to the needs of ophthalmologists, non-specialist clinicians, and patients. Autonomous AI should continue to be evaluated separately from clinician-facing systems because the two models differ in their requirements for oversight, responsibility, and safety.

*From Isolated Applications to Integrated Care Pathways: Enabling Human-Centered Clinical Collaboration:* The second priority is to integrate AI across the complete screening–diagnosis–treatment–follow-up pathway. Multimodal systems should combine imaging, medical history, laboratory findings, and longitudinal data to support not only disease detection, but also treatment timing, therapeutic selection, risk stratification, and individualized surveillance.

Generative AI may assist patient education, clinical documentation, and communication, but its evaluation should report model version, prompting strategy, retrieval sources, hallucination rates, and procedures for professional verification. Workflow design should also define how ungradable, discordant, or uncertain results are transferred to ophthalmologists and how AI outputs are connected to referral tracking, confirmatory assessment, treatment access, and follow-up.

The goal is not simply to insert AI into existing workflows, but to redesign care around complementary roles. AI can provide structured evidence, patients can express their preferences and practical constraints, and physicians can integrate these inputs with clinical judgment. Such integration should reduce cognitive burden and workflow friction while preserving meaningful patient participation and professional responsibility.

*From Initial Deployment to Continuous Clinical Oversight: Advancing Lifecycle Governance and Value-Based Adoption:* The third priority is to manage ophthalmic AI as an evolving clinical technology rather than as software that is validated once and then used indefinitely. Post-deployment monitoring should assess model drift, subgroup performance, image gradability, false-negative events, clinician overrides, referral completion, treatment access, and patient outcomes.

Healthcare institutions should clearly define responsibility for image acquisition, result communication, specialist review, referral initiation, and follow-up. Changes in model performance, intended use, devices, software versions, or patient populations should trigger reassessment rather than being treated as routine technical updates.

Regulatory, procurement, and reimbursement frameworks should also reflect the value of the entire care pathway. Support should extend beyond the AI test itself to include system maintenance, human oversight, patient navigation, safety monitoring, and equitable access. Sustainable adoption will depend on whether AI improves completed care and patient outcomes, rather than merely increasing the number of automated assessments.

As summarized in Figure 6, these three directions represent an iterative progression from trustworthy human-AI evaluation, to integrated patient-centered care pathways, and finally to continuous governance and value-based implementation. Their development should occur in parallel rather than as strictly sequential stages, with evidence from clinical deployment continuously informing technological refinement and policy adjustment. The objective is not maximal automation, but a sustainable model in which AI expands clinical capacity, patients participate more actively in care, and ophthalmologists retain contextual judgment, human connection, and responsibility. Through continued collaboration and iterative progress, we aim to establish a comprehensive, patient-centered paradigm of human-AI collaboration in ophthalmic care by 2035.

We advocate for the establishment of a multidisciplinary consortium comprising medical associations, regulatory agencies, computer scientists, and patient advocates. This coalition should transcend superficial collaboration to codify specific technical standards, clinical guidelines, and ethical frameworks in the domains outlined above. Such a collaborative model represents not merely technological progress but a reaffirmation of medicine’s founding principles—balancing scientific rigor with compassionate care. As AI technologies advance, their ultimate success metric resides in whether they meaningfully augment ophthalmologists’ therapeutic capabilities, thereby making high-quality, equitable eye care accessible to all individuals, irrespective of geography or socioeconomic status. The future of ophthalmology lies not in human-machine competition, but in their harmonious integration—working collectively to preserve one of humanity’s most vital senses: vision.

## 8 Limitations

Several limitations should be considered when interpreting the available evidence. The literature remains unevenly distributed across disease areas and clinical applications, with diabetic retinopathy screening accounting for a substantial proportion of published studies. By comparison, evidence for glaucoma management, age-related macular degeneration, dry eye disease, treatment planning, and long-term follow-up is less developed. Many studies were retrospective, conducted at a single center, or performed in controlled reader-study settings, and external validation across different devices, populations, and healthcare systems was often limited. In addition, most investigations focused on diagnostic accuracy or workflow efficiency rather than long-term visual outcomes, quality of life, sustained adherence, or patient safety. Economic findings were highly dependent on local assumptions and healthcare settings, while acceptance studies may have been affected by selection and response bias. Findings involving large language models should also be interpreted cautiously because performance may vary with model version, prompting strategy, language, and retrieval source.

This review also has methodological limitations. We included only English-language studies indexed in PubMed/MEDLINE, Web of Science Core Collection, and Scopus; therefore, relevant non-English publications and some clinically important engineering or conference literature may not have been captured. The included studies varied considerably in disease area, AI function, study design, clinical setting, and outcome definition, which precluded quantitative pooling and required a narrative synthesis. Mapping the evidence onto perceptual, decisional, and empathetic augmentation also involved interpretive judgment, and some studies contributed to more than one domain. The proposed tripartite framework should therefore be regarded as an evidence-informed organizational model rather than a formally validated classification system. Finally, ophthalmic AI is developing rapidly, and new evidence emerging after the final search date may change the relative maturity of different applications and require future updates of this framework.

## 9 Conclusion

Current evidence supports the concept of the AI-Augmented Ophthalmologist as a model of complementary, rather than substitutive, intelligence in chronic ocular disease care. AI can extend clinical perception, support risk stratification and treatment decisions, and improve the communication and continuity needed for patient participation. The strongest evidence currently comes from diabetic retinopathy screening and referral, where prospective and real-world studies show that timely AI-supported assessment can expand access and improve follow-up. Applications in multimodal diagnosis, treatment planning, longitudinal management, and patient-facing generative AI are developing rapidly, but require further prospective and real-world validation.

The clinical value of this model will depend on more than algorithmic accuracy. Safe and effective implementation requires disease-specific oversight, hybrid pathways for uncertain or ungradable cases, interpretable communication, referral and treatment tracking, local evaluation of resource and economic impact, and continuous monitoring after deployment. Most importantly, AI should be integrated into the complete screening–diagnosis–treatment–follow-up pathway as part of accountable clinical care, rather than used as an isolated replacement for professional judgment.

Accordingly, the future of AI in chronic ocular disease care should be defined by patient-centered human-AI collaboration: AI provides scalable analytical and organizational support, patients contribute their values and preferences, and ophthalmologists retain contextual judgment, human connection, and clinical responsibility. This collaborative paradigm captures the central aim of the AI-Augmented Ophthalmologist framework.
