# Supplementary material for: The AI-Augmented Ophthalmologist for chronic ocular diseases: a patient-centered framework for human-AI collaboration
Source: Front Cell Dev Biol. 2026 Aug 18;14:1902233. doi: 10.3389/fcell.2026.1902233 (PMC13530736; doi:10.3389/fcell.2026.1902233)
Supplement: Supplementary file 1 [file Table1.docx]

**Supplementary Table 1. Database-specific search strategies**

Searches were conducted on July 8, 2026, covering publications from January 1, 2010, to July 8, 2026.

| **Database and search date** | **Search field** | **Complete search strategy** | **Limits and records** |
| --- | --- | --- | --- |
| PubMed/MEDLINE July 8, 2026 | Title/Abstract | (  "diabetic retinopathy"[Title/Abstract]  OR "diabetic macular edema"[Title/Abstract]  OR "diabetic macular oedema"[Title/Abstract]  OR glaucoma[Title/Abstract]  OR "age-related macular degeneration"[Title/Abstract]  OR "geographic atrophy"[Title/Abstract]  OR "neovascular age-related macular degeneration"[Title/Abstract]  OR "neovascular AMD"[Title/Abstract]  OR AMD[Title/Abstract]  OR "dry eye"[Title/Abstract]  OR "dry eye disease"[Title/Abstract]  OR "ocular surface disease"[Title/Abstract] ) AND (  "artificial intelligence"[Title/Abstract]  OR "machine learning"[Title/Abstract]  OR "deep learning"[Title/Abstract]  OR "large language model"[Title/Abstract]  OR "large language models"[Title/Abstract]  OR LLM[Title/Abstract]  OR ChatGPT[Title/Abstract]  OR GPT[Title/Abstract]  OR "generative AI"[Title/Abstract]  OR "foundation model"[Title/Abstract]  OR "foundation models"[Title/Abstract]  OR "clinical decision support"[Title/Abstract]  OR "decision support system"[Title/Abstract]  OR "decision support systems"[Title/Abstract] ) AND (  "AI-assisted"[Title/Abstract]  OR "artificial intelligence-assisted"[Title/Abstract]  OR "AI assisted"[Title/Abstract]  OR "AI-assisted diagnosis"[Title/Abstract]  OR "AI-assisted decision"[Title/Abstract]  OR "AI-assisted screening"[Title/Abstract]  OR "clinical decision support"[Title/Abstract]  OR "decision support"[Title/Abstract]  OR "risk prediction"[Title/Abstract]  OR "risk stratification"[Title/Abstract]  OR triage[Title/Abstract]  OR referral[Title/Abstract]  OR "clinical workflow"[Title/Abstract]  OR "workflow integration"[Title/Abstract]  OR "clinical implementation"[Title/Abstract]  OR implementation[Title/Abstract]  OR "real-world"[Title/Abstract]  OR "reader study"[Title/Abstract]  OR "observer performance"[Title/Abstract]  OR "physician performance"[Title/Abstract]  OR "human-in-the-loop"[Title/Abstract]  OR "human-AI collaboration"[Title/Abstract]  OR "human AI collaboration"[Title/Abstract]  OR "clinician-AI"[Title/Abstract]  OR "physician-AI"[Title/Abstract]  OR "remote monitoring"[Title/Abstract]  OR "home monitoring"[Title/Abstract]  OR adherence[Title/Abstract]  OR compliance[Title/Abstract]  OR "patient engagement"[Title/Abstract]  OR "patient-centered care"[Title/Abstract]  OR "shared decision-making"[Title/Abstract]  OR "health literacy"[Title/Abstract]  OR trust[Title/Abstract]  OR explainability[Title/Abstract]  OR "explainable AI"[Title/Abstract]  OR "automation bias"[Title/Abstract]  OR "over-reliance"[Title/Abstract]  OR overreliance[Title/Abstract]  OR anchoring[Title/Abstract] ) | English 2010/01/01–2026/07/08 Records: 1,120 |
| Web of Science Core Collection July 8, 2026 | Topic (TS) | TS=(  (  "diabetic retinopathy" OR "diabetic macular edema" OR "diabetic macular oedema" OR glaucoma OR "age-related macular degeneration" OR "geographic atrophy" OR "neovascular age-related macular degeneration" OR "neovascular AMD" OR AMD OR "dry eye" OR "dry eye disease" OR "ocular surface disease"  )  AND  (  "artificial intelligence" OR "machine learning" OR "deep learning" OR "large language model" OR "large language models" OR LLM OR ChatGPT OR GPT OR "generative AI" OR "foundation model" OR "foundation models" OR "clinical decision support" OR "decision support system" OR "decision support systems"  )  AND  (  "AI-assisted" OR "artificial intelligence-assisted" OR "AI assisted" OR "AI-assisted diagnosis" OR "AI-assisted decision" OR "AI-assisted screening" OR "clinical decision support" OR "decision support" OR "risk prediction" OR "risk stratification" OR triage OR referral OR "clinical workflow" OR "workflow integration" OR "clinical implementation" OR implementation OR "real-world" OR "reader study" OR "observer performance" OR "physician performance" OR "ophthalmologist performance" OR "human-in-the-loop" OR "human-AI collaboration" OR "human AI collaboration" OR "clinician-AI" OR "physician-AI" OR "remote monitoring" OR "home monitoring" OR adherence OR compliance OR "patient engagement" OR "patient-centered care" OR "shared decision-making" OR "health literacy" OR trust OR explainability OR "explainable AI" OR "automation bias" OR "over-reliance" OR overreliance OR anchoring  ) ) | English; Article 2010–2026 Records: 1,250 |
| Scopus July 8, 2026 | TITLE-ABS-KEY | TITLE-ABS-KEY (  (  "diabetic retinopathy" OR "diabetic macular edema" OR "diabetic macular oedema" OR glaucoma OR "age-related macular degeneration" OR "geographic atrophy" OR "neovascular age-related macular degeneration" OR "neovascular AMD" OR "dry eye disease" OR "ocular surface disease"  )  AND  (  "artificial intelligence" OR "machine learning" OR "deep learning" OR "large language model" OR "large language models" OR ChatGPT OR "generative AI" OR "foundation model" OR "foundation models" OR "clinical decision support" OR "decision support system" OR "decision support systems"  )  AND  (  "AI-assisted diagnosis" OR "AI-assisted decision" OR "AI-assisted screening" OR "clinical decision support" OR "decision support system" OR "human-in-the-loop" OR "human-AI collaboration" OR "clinician-AI" OR "physician-AI" OR "reader study" OR "observer performance" OR "physician performance" OR "ophthalmologist performance" OR "workflow integration" OR "clinical implementation" OR "risk prediction" OR "risk stratification" OR "remote monitoring" OR "home monitoring" OR "patient engagement" OR "patient-centered care" OR "shared decision-making" OR "automation bias" OR "over-reliance" OR overreliance OR anchoring  ) ) AND PUBYEAR > 2009 AND LANGUAGE (english) AND DOCTYPE (ar) | English; Article PUBYEAR > 2009 Records: 520 |
